# Green Adsorbents for Wastewaters: A Critical Review

**DOI:** 10.3390/ma7010333

**Published:** 2014-01-13

**Authors:** George Z. Kyzas, Margaritis Kostoglou

**Affiliations:** 1 Laboratory of General and Inorganic Chemical Technology, Department of Chemistry, Aristotle University of Thessaloniki, Thessaloniki GR-541 24, Greece; E-Mail: kostoglou@chem.auth.gr; 2 Department of Petroleum and Natural Gas Technology, Technological Educational Institute of Kavala, Kavala GR-654 04, Greece

**Keywords:** green adsorbents, wastewaters, agricultural wastes, techno-economical analysis, low-cost materials, dyes, heavy metals, phenols, pesticides

## Abstract

One of the most serious environmental problems is the existence of hazardous and toxic pollutants in industrial wastewaters. The major hindrance is the simultaneous existence of many/different types of pollutants as (i) dyes; (ii) heavy metals; (iii) phenols; (iv) pesticides and (v) pharmaceuticals. Adsorption is considered to be one of the most promising techniques for wastewater treatment over the last decades. The economic crisis of the 2000s led researchers to turn their interest in adsorbent materials with lower cost. In this review article, a new term will be introduced, which is called “green adsorption”. Under this term, it is meant the low-cost materials originated from: (i) agricultural sources and by-products (fruits, vegetables, foods); (ii) agricultural residues and wastes; (iii) low-cost sources from which most complex adsorbents will be produced (*i.e.*, activated carbons after pyrolysis of agricultural sources). These “green adsorbents” are expected to be inferior (regarding their adsorption capacity) to the super-adsorbents of previous literature (complex materials as modified chitosans, activated carbons, structurally-complex inorganic composite materials *etc.*), but their cost-potential makes them competitive. This review is a critical approach to green adsorption, discussing many different (maybe in some occasions doubtful) topics such as: (i) adsorption capacity; (ii) kinetic modeling (given the ultimate target to scale up the batch experimental data to fixed-bed column calculations for designing/optimizing commercial processes) and (iii) critical techno-economical data of green adsorption processes in order to scale-up experiments (from lab to industry) with economic analysis and perspectives of the use of green adsorbents.

## 1. Introduction

Numerous works have been recently published with the primary goal being the investigation of removal of different pollutants (either in gas or liquid medium) using adsorbent materials [1–23]. However, the structure and “philosophy” of adsorbents used are not the same during the years. The latter is due to the change of the under-removal-pollutants. A first obvious change from past to present was the discovery of synthetic dyes, when things began to change. Cheaper to produce, brighter, more color-fast, and easier to apply to fabric are some of the characteristics of these new dyes. Scientists have formulated gorgeous new colors, and synthetic dyes have become obsolete for most applications. No doubt, this bright colored material has changed the world; however, the chemicals used to produce dyes are often toxic, carcinogenic, or even explosive [24]. Among the different pollutants of aquatic ecosystems, dyes are a major group of chemicals [25–28]. Many industries with products such as textiles, leather, cosmetics, paper, printing, plastics, *etc.*, use many synthetic dyes to color their products. Thus, effluents from these industries contain various kinds of synthetic dyestuffs. For instance, dyes used in the textile industries are classified into three classes: (a) anionic (direct, acid, and reactive dyes); (b) cationic (all basic dyes) and (c) non-ionic (dispersed dyes). Basic and reactive dyes are extensively used in the textile industry because of their favorable characteristics of bright color, being easily water soluble, cheaper to produce, and easier to apply to fabric [29–31].

Over 100,000 commercially available dyes exist and more than 700,000 tons are produced annually [32,33]. Due to their good solubility, synthetic dyes are common water pollutants and they may frequently be found in trace quantities in industrial wastewater. An indication of the scale of the problem is given by the fact that two per cent of dyes that are produced are discharged directly in aqueous effluent [32,34]. Due to increasingly stringent restrictions on the organic content of industrial effluents, it is necessary to eliminate dyes from wastewater before it is discharged. Many of these dyes are also toxic and even carcinogenic and this poses a serious hazard to aquatic living organisms [35,36]. However, wastewater containing dyes is difficult to treat without investment (or advanced treatment technique), since the dyes are resistant to aerobic digestion and immune to light, heat and oxidizing agents [37,38].

The most studied dye classes, in the dye bearing effluent treatment, are reactive and basic [39–41]. The dye loss from the dyeing process to the effluent is estimated to be 10%–50% for reactive dyes and 0%–5% for basic ones [42]. Given that reactive and basic dyes could simultaneously exist in the equalization tank of a dye-house, it is of fundamental importance to remove both of them [43]. The research on dyeing wastewater treatment has often focused on reactive dyes for three main reasons: (i) reactive dyes represent an increasing market share, because they are used to dye cotton fibers, which makes up about half of the world’s fiber consumption; (ii) a large fraction, typically around 30% of the applied reactive dyes, is wasted due to the dye hydrolysis in alkaline dye bath and (iii) conventional wastewater treatment plants have a low removal efficiency for reactive and other anionic soluble dyes, which leads to colored waterways [40,44].

The presence of color and color-causing compounds has always been undesirable in water for any use. It is, therefore, not at all surprising to note that the color in wastewater has now been considered as a pollutant that needs to be treated before discharge. Thus, color removal is one of the most difficult challenges to be addressed by textile finishing, dye manufacturing, pulp and paper industries, among others. These industries are major water consumers and are, therefore, a source of considerable pollution. In order to implement an appropriate treatment process, it is of utmost importance to minimize pollution, and to do that, it is necessary to know its exact nature. Robinson *et al*. [34] attempted to give some collective information related to current available technologies and have suggested an effective, cheaper alternative for dye removal and decolorization applicable on a large scale. They have also provided some important data related for desorption of individual textile dyes and a synthetic dye effluent from dye-adsorbed agricultural residues using solvents [45,46], which is also important.

**Figure 1.**
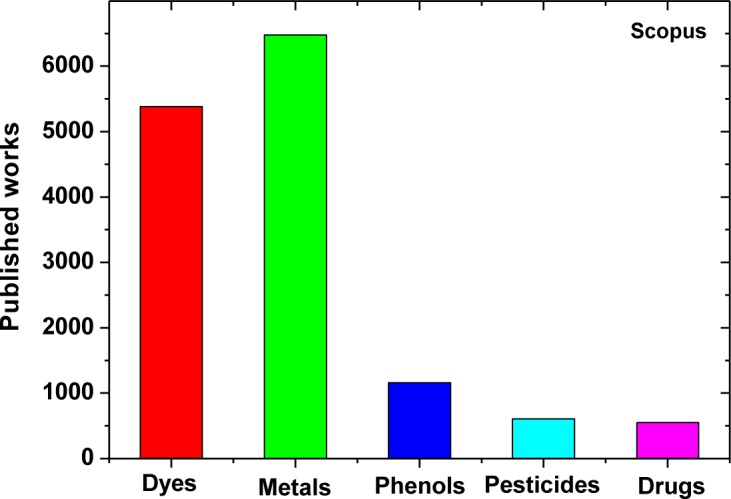
Works published for “adsorption” and various environmental (Data after search in Scopus).

The adsorption technique has become more popular in recent years for wastewater treatment owing to its efficiency in the removal of pollutants too stable for biological methods (Figure 1). Dye adsorption is a result of two mechanisms (adsorption and ion exchange) and is influenced by many factors such as dye/adsorbent interaction, adsorbent’s surface area, particle size, temperature, pH and contact time. The main advantage of adsorption recently became the use of low-cost materials, which reduces the procedure cost.

However, following the economic boom in the 1970s—where the procedure cost was not such a big problem (or had not been taken into account)—the economic crisis of the 2000s arose and led researchers to turn their interest to other fields with lower procedure costs. Therefore, different materials were tested as possible wastewater adsorbents. The selection for the most appropriate adsorbent would be based on some major characteristic properties such as: (i) the low-cost along with the satisfactory adsorption properties (capacity, reuse, industrial-scale use *etc*.) and (ii) the environmentally-friendly nature of each adsorbent. It is fundamental to use only materials which either have the lowest impact on environmental balance or are absolutely environmentally-friendly (from abundant natural sources, biodegradable, non-toxic *etc.*).

Therefore, a new term will be introduced in this study, which is called “green adsorption”. Under this term, it is meant that the low-cost materials originated from: (i) agricultural sources and by-products (fruits, vegetables, foods); (ii) agricultural residues and wastes and (iii) low-cost sources from, which most complex adsorbents will be produced (*i.e.*, activated carbons after pyrolysis of agricultural sources). These “green adsorbents” will be expected to be inferior (regarding their adsorption capacity) than the super-adsorbents of literature (complex materials as modified chitosans, activated carbons, structurally-complex inorganic composite materials *etc.*), but their cost-potential makes them competitive.

The present review is not a repetition of the up-to-now literature. It is a critical approach to green adsorption, discussing many different (maybe in some occasions doubtful) topics such as: (i) the principal adsorption parameter of each material which is its capacity (mass of pollutant adsorbed onto adsorbent per adsorbent’s mass); (ii) the kinetic model/equation used for the description of adsorption procedure which is a very serious factor, regarding the ultimate target to scale up the batch experimental data to fixed-bed column calculations for designing/optimizing commercial processes; and (iii) attempt to adjust some crucial techno-economical data of adsorption process in order to scale-up experiments (from lab to industry) with possible economic analysis and perspectives of the use of green adsorbents. As environmental pollutants, two basic categories were employed in the present review (dyes and heavy metals) as well as some other (pharmaceuticals, pesticides, phenols), which despite their limited research investigation, they existed in wastewaters. It is fact that apart from the above topics, there is much to be discussed in regards to characterization results and data from green adsorbents, synthesis/fabrication of them, their structural characteristics, *etc.* However, in that case, the main focus of this review article would be changed. Therefore, we have chosen to analyze in detail the three main topics (capacity, kinetics, techno-economical data).

## 2. Adsorption Capacity

The key-point of each adsorbent material is its adsorption capacity. Three isotherm models are given in recent literature in order to fit the experimental equilibrium data: the Langmuir equation Equation (1) [47], the Freundlich equation Equation (2) [48] and the combinational Langmuir-Freundlich (L-F) equation Equation (3) isotherm model [49]:

$$Q_{e}=\frac{Q_{m}K_{L}C_{e}}{1+K_{L}C_{e}}$$ (1)

$$Q_{e}\text{= }K_{F}C_{e}{}^{1\text{/}n}$$ (2)

$$Q_{e}\text{ = }\frac{Q_{m}K_{\text{LF}}{\left(C_{e}\right)}^{\text{1/}b}}{1+K_{\text{LF}}{\left(C_{e}\right)}^{\text{1/}b}}$$ (3)

where *Q*_e_ (mg/g) is the equilibrium concentration of pollutant in the solid phase; *Q*_m_ (mg/g) is the maximum amount of adsorption; K_L_ (L/mg) is the Langmuir adsorption equilibrium constant; K_F_ (mg^1−1/n^·L^1/n^/g) is the Freundlich constant representing the adsorption capacity; n (dimensionless) is the constant depicting the adsorption intensity; K_LF_ (L/mg)^1/b^ is the Langmuir-Freundlich constant; and b (dimensionless) is the Langmuir-Freundlich heterogeneity constant.

The amount of total uptake of pollutant in equilibrium (*Q*_e_) was calculated using the mass balance equation Equation (4):

$$Q_{e}=\frac{(C_{0}-C_{e})V}{M}$$ (4)

where *M* (g) is the mass of adsorbent; *V* (L) the volume of adsorbate; *C*_0_ and *C*_e_ (mg/L) are the initial and equilibrium concentrations of pollutant in the liquid phase, respectively.

In this study, in order to avoid repeating the up-to-now literature, it will not be discussed how appropriate a particular isotherm model is for an adsorbent-adsorbate system. These reports (and especially review articles) have already been extensively discussed in other works [24,50–54]. However, a discussion will be had regarding the adsorption capacity of each green adsorbent. In this review, the most appropriate classification is based on the pollutants removed. However, another classification could be realized based on adsorbents and insights on structural relevancy or their main structural conversion strategies. However, in that case, the scope of this study would be changed.

### 2.1. Dyes

Methylene Blue (MB) is the most commonly used dyestuff for the dying of wool and silk. It may cause eye burns which may be responsible for permanent injury to the eyes of human and animals. On inhalation, it can give rise to short periods of rapid or difficult breathing while ingestion through the mouth produces a burning sensation and may cause nausea, vomiting, profuse sweating, mental confusion and methemoglobinemia [55,56]. Therefore, the treatment of effluent containing such dye is of interest due to its harmful impacts on receiving waters.

Chatterjee and co-workers [57] studied the adsorption removal of MB using a low-cost adsorbent, prepared from *Parthenium hysterophorus. Parthenium* (a harmful weed) is carbonized to produce low-cost adsorbent using ortho-phosphoric acid as activating agent. The fitting was done with Langmuir (*R*^2^ = 0.99) and Freundlich equation (*R*^2^ = 0.95). The *Q*_m_ of this process was 98.06 mg/g, while authors claimed that this values was competitive to other similar-based adsorbents regarding MB removal as: 89.4 mg/g of rectorite [58], 60.6 mg/g of acid activated carbon prepared from morinda coreia buch-ham bark [59], 39.68 mg/g of adsorbent prepared from parthenium [60], 24 mg/g of adsorbent prepared from Denix regia pods [61]. Barka *et al*. [62] investigated the biosorption of some dyes [MB, Eriochrome Black T (EBT) and Alizarin S (AS)] from aqueous solutions using dried prickly pear cactus cladodes as a low-cost, natural and eco-friendly biosorbents. Their *Q*_m_ (in particular for MB) were much more higher than those of Chatterjee *et al*. [57]. The maximum monolayer adsorption capacities were 189.83 mg/g for MB, 200.22 mg/g for EBT and 118.35 mg/g for AS. On the other hand, Ferrero [63] studied the adsorption of MB onto ground hazelnut shells in comparison with sawdust of various species of wood, in order to explore the potential use of this material as green adsorbent for dyehouse effluents. A very interesting finding was that *Q*_m_ (MB) for hazelnut shells (*d*_p_ = 500 μm) was 41.3 mg/g, which was five times higher than the respective amount reported for activated carbon obtained from the same material. The same value for *d*_p_ = 125 μm was 76.9 mg/g. Hameed [64] investigated the feasibility of using papaya seeds—which are abundantly available wastes in Malaysia—for MB adsorption with a high adsorption capacity (556 mg/g). McKay and co-workers [65] found high adsorption capacities for MB and Safrarine (basic dye) using some very strange low-cost materials as green adsorbents (*i.e.*, cotton waste, hair from barber shop *etc.*). The *Q*_m_ for Safranine were 1119, 838, 875, 190 and 120 mg/g and for MB were 914, 312, 277, 158 and 250 mg/g for tea wood bark, rice husk, cotton waste, hair and bituminous coal, respectively.

Another major cationic dye of wastewaters is Malachite Green (MG). MG is an organic compound which is used as dyestuff and has emerged as a controversial agent in aquaculture. MG is traditionally used as a dye for materials such as silk, leather, and paper [40]. Many different papers were published with different and in some cases very doubtful capacities due to the extremely high deviation observed. In particular, Iqbal and co-workers [66] found *Q*_m_ = 0.179 mg/g for the adsorption onto activated charcoal. This extremely low capacity could characterize this material as completely inappropriate. Tahir *et al*. [67] showed a value of *Q*_m_ equal to 7.72 mg/g for MG adsorption onto bentonite clay. Activated carbons of commercial grade (ACC) similarly presented low *Q*_m_ (8.27 mg/g) [68], as well as sugar cane dust (4.88 mg/g) [69] and hen feathers (26.1 mg/g) [70]. Other studies showed higher *Q*_m_, as in the case of using lemon peels as green adsorbents (51.73 mg/g) [71] or coconut coir activated carbon (27.44 mg/g) [72].

El Haddad and co-workers [73] studied the adsorption removal of Rhodamine B from wastewaters with animal bone meal as a new green adsorbent. The capacities obtained at different temperatures were close to 65 mg/g. Vijaya Kumar [74] studied the adsorption of Violet 54 (V54) using a musa spp. waste adsorbent, but the *Q*_m_ was found to be low (36.49 mg/g). Orange peels were also tested as green adsorbents for Direct Red 23 (DR23) and Direct Red 80 (DR80) by Doulati Ardejani *et al*. [75], but low capacities were shown (10 and 21 mg/g, respectively). Some disperse dyes (their commercial names are Begacron Blue BBLS 200% and Miketon Polyester Scarlet RCS) were removed with palm ash as presented by Hasnain Isa and co-workers [76], but similarly in this study the *Q*_m_ were low enough (49.5 and 61 mg/g, respectively). El-Mekkawi and Galal [77] investigated that the adsorption capacity of rutile TiO_2_ and Degussa P25 TiO_2_ for the removal of Direct Fast Blue B2RL were 56 and 144 mg/g (optimum pH = 2), respectively.

A first comment on the above is the complete unevenness of the adsorption capacities found. It is fact that the literature is chaotic, but a first statistic result was clear. There were green adsorbents with high *Q*_m_ of over 150 mg/g, while some others had a extremely low capacity (<15 mg/g).

### 2.2. Metals-Ions

Interesting works have been published regarding the adsorption of various heavy metals onto green adsorbents. Cochrane and co-workers [78] investigated the use of three biosorbents (crab carapace, macroalgae *Fucus vesiculosus*, peat) for the removal of copper from aqueous media. The results were directly compared with two commercial materials (activated carbon and ion-exchange resin). Langmuir and Freundlich isotherms were used to describe the adsorption equilibrium data. The *Q*_m_ values were 79.4, 114.9 and 71.4 mg/g for crab carapace, *F. vesiculosus* and ion-exchange resin, respectively.

A recent study of Abdel Salam and co-workers [79] showed the adsorption behavior of some low-cost adsorbents such as peanut husk charcoal, fly ash, and natural zeolite, with respect to copper and zinc ions, in order to consider their application to the purification of metal finishing wastewater. Adsorption parameters were determined using both Langmuir and Freundlich isotherms, but the experimental data were better fitted to the Langmuir equation than to Freundlich equation. The results showed that peanut husk charcoal, fly ash and natural zeolite all hold potential to remove cationic heavy metal species from industrial wastewater in the following order: fly ash (0.18 mg/g) < peanut husk charcoal (0.36 mg/g) < natural zeolite (1.18 mg/g). However, the *Q*_m_ values were extremely low and could not be attractive, not only for batch experiments but also for industrial use.

The removal of Cr(VI) from aqueous solution with batch adsorption techniques using different low-cost adsorbents was investigated by Bhattacharya *et al*. [80]. He used some low-cost adsorbents such as clarified sludge (a steel industry waste material), rice husk ash, activated alumina, fuller’s earth, fly ash, saw dust and neem bark to determine the adsorption efficiency for Cr(VI). Langmuir model perfectly fitted the equilibrium data (*R*^2^ ~ 0.999), but demonstrated low adsorption capacities (19–31 mg/g) as: clarified sludge (26.31 mg/g), rice husk ash (25.64 mg/g), activated alumina (25.57 mg/g), fuller’s earth (23.58 mg/g), fly ash (23.86 mg/g), saw dust (20.70 mg/g) and neem bark (19.60 mg/g).

A strange adsorbent material (straw) was used by Kumar and co-workers [81] in order to remove heavy metals from aqueous systems. The straw was initially modified to alkali-treated straw (ATS) and insoluble straw xanthate (ISX), which slightly increased the cost of adsorbent. The *Q*_m_ for the removal of Cr(III) was very low (1.88 and 3.91 mg/g for ATS and ISX, respectively).

Aziz and co-workers [82] investigated the adsorption of cadmium from treated olive stones (TOS) and after Langmuir modeling the calculated *Q*_m_ was 49.3 mg/g.

Heavy metals such as Cr(III), Cu(II) and Zn(II) were able to be removed from wastewater using HCl treated carrot residues. Acid treatment was performed in order to remove tannins, resins, reducing sugars and coloured materials. According to Nasernejad and co-workers [83], adsorption of metal ions onto carrot residues was possible due to the presence of carboxylic and phenolic groups which have cation exchange properties. More metals were adsorbed at higher pH values of the solutions (pH 4 for Cr(III) and pH 5 for Cu(II) and Zn(II)). Maximum adsorption capacities were 45.09, 32.74 and 29.61 mg/g for Cr(III), Cu(II) and Zn(II), respectively.

Perez-Marin *et al*. showed that the untreated orange waste could only adsorb 48.33 mg/g Cd(II) [84]. Previously, Wartelle and Marshall [85] reported an interesting finding in which a linear relationship between total negative charge and amount of copper ions adsorbed was observed for 12 types of agricultural byproducts (sugarcane bagasse, peanut shells, macadamia nut hulls, rice hulls, cottonseed hulls, corn cob, soybean hulls, almond shells, almond hulls, pecan shells, English walnut shells and black walnut shells) after modification with citric acid. It was found that after washing with base (NaOH) and modified with citric acid, the total negative charge of all 12 types of agricultural by-products increased significantly. Among the 12 adsorbents, soybean hulls (a low density material) showed the highest copper uptake and had a high total negative charge value, which can be explained by the increase in carboxyl groups after thermochemical reaction with citric acid. On the other hand, nutshells (high density materials) such as English walnut shells and black walnut shells displayed low total negative charge values indicating a low number of carboxyl groups. Due to the high bulk density, the lignin in nutshells may block or allow little penetration of citric acid to reactive sites; hence, lower copper ion uptake was observed.

Azolla, a small aquatic fern, is commonly used as fertilizer in botanical gardens and as green manure in rice fields. Binding or ion-exchange of heavy metal ions is possible due to the presence of charged groups such as carboxyl and phosphate in the Azolla matrix [86]. The percentage adsorption values of Pb, Cd, Cu and Zn by Azolla treated with MgCl_2_ alone were approximately 33, 29, 40 and 24, respectively. These values however increased with increasing concentration of MgCl_2_ due to better ion exchange behavior between heavy metals and Mg^2+^ ions on the cell walls of Azolla. No remarkable effect on the heavy metal removal was observed when Azolla was treated with H_2_O_2_ alone. However, the highest metal removal was reported by treating Azolla with 2 M MgCl_2_ in the presence of 8 mM H_2_O_2_. The maximum adsorption capacities for Pb(II), Cd(II), Cu(II) and Zn(II) were 228, 86, 62 and 48 mg/g, respectively.

Adsorption of divalent heavy metal ions particularly Cu^2+^, Zn^2+^, Co^2+^, Ni^2+^ and Pb^2+^ onto acid and alkali treated banana and orange peels was performed by Annadurai and co-workers [87]. The acid and alkali solutions used for modification of adsorbents were HNO_3_ and NaOH. In general, the adsorption capacity decreases in the order of Pb^2+^ > Ni^2+^ > Zn^2+^ > Cu^2+^ > Co^2+^ for both adsorbents. Banana peel exhibits higher maximum adsorption capacity for heavy metals compared to orange peel. The reported maximum adsorption capacities were 7.97 (Pb), 6.88 (Ni), 5.80 (Zn), 4.75 (Cu) and 2.55 mg/g (Co) using banana peel; and were 7.75 (Pb), 6.01 (Ni), 5.25 (Zn), 3.65 (Cu) and 1.82 mg/g (Co) using orange peel. Acid treated peels showed better adsorption capacities followed by alkali and water treated peels. Based on regeneration studies, it was reported that the peels could be used for two regenerations for removal and recovery of heavy metal ions.

A special comment should be done regarding the analysis of heavy metals in wastewaters. The up-to-now method for metal analysis after adsorption is indirect. A volume of the adsorbate is collected and analyzed with AAS (atomic absorption spectroscopy) or more recently ICP-MS (inductively coupled plasma mass spectrometry). So, there is no special need to measure the heavy metal concentration with some ashing procedure onto materials (direct method).

### 2.3. Others

Phenols are the major part of other pollutants studied using green adsorbents. Namasivayam and Kavitha [88] investigated the adsorption of 2-chlorophenol (2-CP) by coir pith carbon by varying some parameters as agitation time, phenol’s concentration, adsorbent’s dosage, pH and temperature. The fitting was done with Freundlich, Langmuir and Dubinin-Radushkevich equations, but only the equilibrium adsorption capacity (*Q*_e_) was presented and not the theoretical maximum one after modeling (*Q*_m_).

Other type of phenols (parachlorophenol (PCP) and 2,4,6-trichlorophenol (TCP)) was studied by Radhika and Palanivelu [89]. The green adsorbent used was a type of activated carbon prepared from coconut shells (CSAC), using some chemical agents (KOH, NaOH, CaCO_3_, H_3_PO_4_ and ZnCl_2_) for differentiating the activation method. Langmuir, Freundlich, Redlich-Peterson and Sips isotherm models fitted the equilibrium data and compared to results from commercial activated carbon (CAC). The best fitting was achieved with the Freundlich model. The *Q*_m_ for PCP was 72 mg/g, which was approximately half compared to the capacity presented from CAC (~150 mg/g). The respective value was higher for TCP (CSAC, 122 mg/g; CAC, 112 mg/g).

A comparative study on the adsorption of methylphenols on adsorbents prepared from several industrial wastes has been carried out by Jain and co-workers [90]. The results showed that extent of adsorption on carbonaceous adsorbent prepared from fertilizer industry waste has been found to be 37.3, 40.5, 65.9, and 88.5 mg/g for 2-methylphenol, 4-methylphenol, 2,4-dimethylphenol, and 2,4,6-trimethylphenol, respectively. As compared to carbonaceous adsorbent, the other three adsorbents (blast furnace sludge, dust, and slag) adsorbed methylphenols to a much smaller extent.

A similar study of Bhatnagar [91] comparatively studied some adsorbents prepared from several industrial wastes for the removal of 2-bromophenol (2-BP), 4-bromophenol (4-BP) and 2,4-dibromophenol (2,4-BP). The results showed that maximum adsorption on carbonaceous adsorbent prepared from fertilizer industry waste has been found to be 40.7, 170.4 and 190.2 mg/g for 4-BP, 2-BP and 2,4-BP, respectively. As compared to carbonaceous adsorbent, the other three adsorbents (blast furnace sludge, dust, and slag) adsorbed bromophenols to a much smaller extent. That fact has been attributed to the carbonaceous adsorbent having a larger porosity and consequently higher surface area.

Mohan *et al*. [92] used activated carbons from coconut in order to remove pyridine from effluents. In particular, FAC (activated carbon derived from coconut fibers), SAC (activated carbon derived from coconut shells), ATFAC (activated carbon derived from acid treated coconut fibers) and ATSAC (activated carbon derived from acid treated coconut shells) were prepared as adsorbents. Langmuir isotherm presented the best fitting (*R*^2^ > 0.9553) giving *Q*_m_: FAC, 20.31 mg/g; SAC, 19.46 mg/g; ATFAC; 54.63 mg/g; ATSAC, 60.35 mg/g; ACF (activated carbon from fabric cloth), 161.40 mg/g.

Another pollutant removed from wastewaters was nitrobenze using maize and rice stems [93]. The latter are two abundant, low-cost crop castoffs which are widely available in China. The equilibrium data were fitted to Langmuir and linearized Freundlich model. Maize stem showed *Q*_m_ equal to 10.40 mg/g, while rice stems much lower (1.31 mg/g). It is not acceptable to consider or use a green adsorbent, which presents such low capacity. Therefore, despite its zero cost, it would be rejected for further consideration.

CuFe_2_O_4_/sawdust nanocomposite was prepared as potential green adsorbent for the removal of cyanine acid blue (CAB) from wastewaters [94]. The sawdust was collected from sawmill in Farrokhi city (Iran). The Langmuir and Freundlich models were tested for predicting the maximum adsorption capacity. The composite showed *Q*_m_ equal to 178.56 mg/g, while using only sawdust the respective value was 151.45 mg/g. An enhancement was observed, but it was uncertain if the whole synthesis of composite was profitable.

The adsorptive potential of selected agricultural waste materials *i.e.*, rice (Oryza sativa) bran (RB), bagasse fly ash (BFA) of sugarcane (*Saccharum officinarum*), *Moringa oleifera* pods (MOP) and rice husk (RH) for the removal of methyl parathion pesticide (MP) from surface and ground waters has been investigated by Akhtar and co-workers [95]. The equilibrium data were fitted to Freundlich, Langmuir and Dubinin-Radushkevich isotherms. The maximum capacities of RB, BFA, MOP and RH for MP were 0.39, 0.39, 0.36 and 0.35 mmol/g (Langmuir equation), respectively.

A few works showed high *Q*_m_ for the removal of pesticides from effluents with low-cost adsorbents. In particular, El Bakouri and co-workers [96] used acid-treated olive stones as green adsorbents in order to remove different pesticides (aldrin, dieldrin, endrin) with low adsorbent’s dosage (0.1 g/L). The *Q*_m_ were 19.54, 23.74 and 43.71 mg/g, respectively. The removal of parquet (pesticide) was also expanded with use of methacrylic acid modified rice husk as adsorbent (292.5 mg/g) [97].

## 3. Kinetics

The term “modeling” in physicochemical processes study is quite general and ranged from simple fitting of the experimental data [98] to detailed models based on basic principles [49]. Especially, in the field of adsorption, quite different modeling approaches can be found. In the present review, some adsorption models (in particular, those which were used for modeling liquid-phase adsorption by low-cost adsorbents) will be presented and discussed. The models will be critically reviewed in this subsection and they will then be simply cited in the discussion of the works using them.

The typical experimental setup includes an initial concentration of adsorbate (*C*_0_), a beaker volume (*V*) and a mass of adsorbent (*M*). The amount of adsorbate adsorbed on the adsorbent (per unit adsorbent mass) is expressed as “*Q*” and evolves from *Q* = 0 to an equilibrium value *Q* = *Q*_e_, which corresponds to the thermodynamic equilibrium between *Q*_e_ and $C_{e}=C_{0}-M\left(\frac{Q}{V}\right)$. The modeling issue is focused on the quantitative description of the evolution curve *Q*(*t*), which is deduced by measuring experimentally the evolution of adsorbate concentration *C*(*t*) from *C*_0_ to *C*_e_. The most extensively used models are typically those with the least physical background. The adsorption kinetic models which have been employed in green adsorption are:

(i) The so-called Lagergren equation [99] (or pseudo-first order model) (abbreviated hereafter as PS1) has the following differential form:

$$\frac{dQ}{dt}=k_{1}\left(Q-Q_{e}\right)$$ (5)

After integration of Equation (5):

$$Q=Q_{e}\left(1-e^{-k_{1}t}\right)$$ (6)

The fitting to the experimental data can be performed using linear (*i.e.*, using $\ln\left(1-\left(Q/Q_{e}\right)\right)=-k_{1}t)$ or nonlinear [*i.e.*, using directly Equation (6)] techniques. The model is purely empirical and the only physical parameter used is the equilibrium value (*Q*_e_). The only fitting parameter is k_1_. Typically, instead of using the experimental value of *Q*_e_ and find k_1_ from fitting, both k_1_ and *Q*_e_ are found from fitting and then *Q*_e_ is compared to the experimental one.

(ii) The second model used is also empirical and it usually gives better fitting results than the first model. It is called pseudo-second order model [100] (abbreviated hereafter as PS2) and has the following governing expression:

$$\frac{dQ}{dt}=k_{2}{\left(Q-Q_{e}\right)}^{2}$$ (7)

After integration of Equation (7):

$$Q=\frac{k_{2}Q_{e}{}^{2}t}{1+k_{2}Q_{e}t}$$ (8)

There are several ways to transform the relation to a linear one in order to use linear fitting techniques. The most usual is:

$$\frac{t}{Q}=\frac{1}{k_{2}Q_{e}{}^{2}}+\frac{t}{Q_{e}}$$ (9)

As in the case of the first-order model, the fitting can be done either for k_2_ alone using the experimental value of *Q*_e_ or for the pair (k_2_, *Q*_e_). The above models are used to find the coefficients k_1_ and k_2_, which are regarded as pure fitting parameters and subsequently depends on operational variables as *C*_0_.

(iii) The third model is the so-called Elovich model [101] (abbreviated hereafter as ELV), which assumes a logarithmic time dependence of the adsorbed species quantity. The governing equation in its linearized form is:

$$Q=\frac{\ln\left(\alpha \beta \right)+\ln\left(t\right)}{\beta }$$ (10)

where the constants α, β are called initial adsorption and desorption rate constants, respectively. Alternatively, α is related to the rate of chemisorption and β to the surface coverage [102]. This equation has typically a poor success in fitting the data but, despite this, it is used extensively in the literature.

(iv) Another model is the intraparticle diffusion model (abbreviated hereafter as INTD). This model is based on the assumption that the adsorption kinetics is dominated by the intraparticle diffusion mechanism [103]. The corresponding equation can be derived for linear isotherm and constant adsorbate concentration, but it is used in literature for much more general conditions:

$$Q=Q_{e}\left(1-\frac{6}{\pi^{2}}\sum_{i=1}^{\infty }\frac{1}{i^{2}}\text{exp}\left(-\frac{Di^{2}\pi^{2}}{R^{2}}t\right)\right)$$ (11)

where *D* is the intraparticle diffusion coefficient and *R* the particle radius. The procedure of fitting Equation (11) to the kinetic experimental data can be found under the name Reichenberg [104] or Helffrich [105] model.

For *Q* far from the equilibrium, the above relation can be simplified to $Q=k_{1D}t^{0.5}$ (where k_1D_ is the intraparticle diffusion constant [106] (abbreviated hereafter as INTD-1). In some cases, the alternative coefficient $k_{\text{in}}=k_{1D}/M$ appears defined as the initial rate of intraparticle diffusion [78]. The last equation is actually more general than Equation (11), since it is restricted only by the smallness of (*Q*/*Q*_e_) and not by the form of isotherm and the constancy of bulk concentration. The model is a phenomenological one, since includes parameters with physical meaning. The relation between k_1D_ and *D* is $k_{1D}=\frac{6}{R{\left(D/\pi \right)}^{0.5}}$. The obvious drawback is that INTD-1 cannot predict the long term equilibrium, being a continuously increasing function of time. Another purely empirical extension of INTD-1 is the addition of a constant value (p) to its right hand side (abbreviated hereafter as INTD-2). This can help to the overall curve fitting process but is has no physical meaning since it implies that *Q* = p at *t* = 0, whereas the real value is *Q* = 0.

(v) The other model is the so-called McKay model [107] (abbreviated hereafter as MCK). This mass transfer model is phenomenological and was derived for the case of a linear isotherm and for mass transfer from the solution to the particle surface being the dominant (slowest) step of the adsorption process. The corresponding equation for the evolution of bulk concentration is (modified to admit use of linear fitting procedures):

$$\ln\left(\frac{C}{C_{0}}-\frac{1}{1+KM}\right)=\ln\left(\frac{KM}{1+KM}\right)-\left(\frac{1+KM}{KM}\right)hSt$$ (12)

where *S* is the total adsorbent particles’ external area; *h* is the mass transfer coefficient and K is the linear isotherm constant. This model was derived in order to analyze the effect of stirring on adsorption kinetics and is meaningful only if the stirring is low enough to dominate the adsorption kinetics. Interestingly enough, the model is used to analyze typical adsorption experimental data taken using high stirring intensity needed for optimization of the global kinetics. Obviously the use of this model in those cases is quite erroneous.

(vi) The chemical reaction engineering approach (abbreviated hereafter as GREA) to adsorption dynamics applies the classical concept of chemical engineering to the adsorption process [49]. The key aspects are that (i) all parameters are of fundamental value; and (ii) these parameters characterize the adsorbate and the adsorbent separately. Unlike the previous models, the present model can be scaled-up in order to be used under different experimental conditions The adsorbate can be found in the adsorbent particle phase as solute in the liquid filling the pores of the particle (concentration *C* in kg/m^3^) and adsorbed on the solid phase (concentration *q* in kg adsorbate/kg adsorbent). The “homogeneous” equations for the evolution of *C* and *Q* inside a spherical adsorbent particle of radius *R* are the following:

$$\varepsilon_{p}\left(\frac{\partial C}{\partial t}\right)=\left(\frac{1}{r^{2}}\right)\left(\frac{\partial }{\partial r}\right)r^{\text{2}}D_{p}\left(\frac{\partial C}{\partial r}\right)-\rho_{p}G\left(C,Q\right)$$ (13)

$$\frac{\partial Q}{\partial t}=\left(\frac{1}{r^{\text{2}}}\right)\left(\frac{\partial }{\partial r}\right)r^{2}D_{s}\left(\frac{\partial Q}{\partial r}\right)-G\left(C,Q\right)$$ (14)

where *r* is the radial direction; ε_p_ the porosity of the particle; *ρ*_p_ the density of the particle (kg adsorbent/m^3^); *D*_p_ is the liquid-phase diffusivity of the adsorbate and *D*_s_ is the corresponding surface diffusivity. The diffusivity *D*_p_ for a given pair of adsorbate-fluid depends only on the temperature, whereas the diffusivity *D*_s_ depends both on the type of adsorbent and on *Q* also (apart from their dependence on geometry). The function *G*(*C*,*Q*) denotes the intrinsic rate of the adsorption-desorption process.

The boundary conditions for the above set of equations are the mass transfer from the solution to the particle (where *C*_b_ is the concentration of the adsorbate in the bulk solution) Equation (15) and spherical symmetry Equation (16)

$$h\left(C_{b}-C\right)=-D_{p}{\left(\frac{\partial C}{\partial r}\right)}_{r=R}$$ (15)

$$\left(\frac{\partial C}{\partial r}\right)=\left(\frac{\partial Q}{\partial r}\right)=0$$ (16)

Having values for the initial concentrations of adsorbate in the particle and for the bulk concentration *C*_b_, the above mathematical problem can be solved for the functions *C*(*r*,*t*) and *Q*(*r*,*t*). The above model is the so-called non-equilibrium adsorption model. In the case of adsorption by small particles, the adsorption-desorption process is much faster than the diffusion, leading to an establishment of a local equilibrium [which can be found by setting *G*(*C*,*Q*) = 0 and corresponds to the adsorption isotherm *Q* = f(*C*)]. In the limit of very fast adsorption-desorption kinetics, it can be shown by a rigorous derivation that the mathematical problem can be transformed to the following:

$$\frac{\partial Q}{\partial t}=\left(\frac{1}{r^{2}}\right)\frac{\partial }{\partial r}r^{2}D\left(C\right)\frac{\partial Q}{\partial r}$$ (17)

$${\left(\frac{\partial Q}{\partial r}\right)}_{r=0}=0$$ (18)

$$h\left(C_{b}-C\right)=\rho_{p}D_{p}{\left(\frac{\partial Q}{\partial r}\right)}_{r=R}$$ (19)

An additional assumption considered in the above derivation is that the amount of the adsorbate found in the liquid phase in the pores of the particle is insignificant compared with the respective amount adsorbed on the solid phase (*i.e.*, ε_p_*C* << ρ_p_*Q*). This assumption always holds, as it can be easily checked by recalling the definition of the adsorption process. The concentration *C* in Equations (17) and (19) can be found by inverting the relation *Q* = f(*C*). The diffusivity *D* is an overall diffusivity, which combines the bulk and surface diffusivities and is given by the relation:

$$D=D_{s}+\frac{D_{p}}{\rho_{p}f^{\prime }\left(C\right)}$$ (20)

where the prime denotes the differentiation of a function with respect to its argument. The average concentration of the adsorbed species can be computed by the relation:

$$Q_{\text{ave}}=\frac{3}{R^{3}}\int_{0}^{R}Qr^{2}dr$$ (21)

In the case of batch experiments, the concentration of the solute in bulk liquid *C*_b_ decreases due to its adsorption; so, the evolution of the *C*_b_ must be taken into account by the model. The easier way to do this is to consider a global mass balance of the adsorbate:

$$C_{b}=C_{0}-\left(\frac{M}{V}\right)Q_{\text{ave}}$$ (22)

(vii) The GREA has the obvious drawback of mathematical complexity (*i.e.*, it is comprised from partial differential equations) whereas all the other models presented above not only comprised from analytical relations but they can be cast in linear form. In order to make simpler GREA without sacrificing its parametric dependency, several techniques of mathematical simplification have been proposed. Typically, the partial differential equation is transformed to a system of up to three ordinary differential equations [108]. A way to achieve this is through a collocation procedure [109]. Another way is to assume a polynomial profile for the unknown function and to attempt to derive an ordinary differential equation for the total adsorbate amount in the adsorbent particle [110]. This procedure can lead to an explicit result for a linear isotherm. In the case of nonlinear isotherms, further assumptions about averaging nonlinear terms are needed. The final result is an ordinary differential equation which retains all the parametric dependency of the original GREA (abbreviated hereafter as ODGREA). The derived equation in the absence of external mass transfer resistance is [where *Q*_e_ = f(*C*_b_) and *Q* = f(*C*)]:

$$\frac{dQ}{dt}=\frac{15}{R^{2}}\left(D_{s}+\frac{D_{p}}{\rho_{p}f^{\prime }\left(C\right)}\right)\left(Q_{e}-Q\right)$$ (23)

The models presented above are selected because they appear in the works that will be reviewed below. They are not the only models but there are many others which are mainly modifications of those discussed above. To name a few: Langmuir kinetic model [111], shrinking core model [103], reversible and irreversible reactions model [112,113], pseudo-n order [114], two site pseudo second order [115], mixed 1,2-order equation [116], mixed diffusion-reaction model [117], modified pseudo-first or -second order [118,119], fractal like modifications [120], *etc.*

All phenomenological models can be cast in terms of the GREA approach. For example, the Langmuir kinetic model can be derived giving the appropriate form in the function *G*(*C*,*Q*). The intraparticle and external mass transfer models are also included in GREA. Only the empirical models PS1 and PS2 seem to be outside the general framework of GREA. Nevertheless, it was shown that pseudo-first order can be considered to emerge from Langmuir kinetic model for a small reduction of the bulk solute concentration [121]. In the same way, the pseudo-second order model can be derived from Langmuir kinetic model under specific conditions [121]. In this way, the empirical models PS1 and PS2 recast into phenomenological approaches and the parameters *k*_1_ and *k*_2_ were related to the physical problem parameters [121]. It is noted that PS2 performs well even in cases where the adsorption is dominated by diffusion and the isotherm is not of Langmuir type so the recast of the model in terms of the Langmuir kinetic model needs further study. In any case, all the presented models can be claimed to belong to the GREA approach.

### 3.1. Dyes

In a work of Chatterjee [57], nine sets of kinetic data using a low cost adsorbent were presented. In particular, three adsorbent dosages, three adsorbate concentrations and three adsorbent particle sizes were considered. PS1, PS2, and INTD-1 were used to fit the data. According to authors, the analysis showed that PS2 fitted the data more satisfactorily than any other model. Nevertheless, no fitting results for the other models were shown. Five kinetic data sets for coconut coir dust as adsorbent for five adsorbate concentrations were presented by Etim and co-workers [122], where PS1 and PS2 were considered. PS2 led to perfect fit with *R*^2^ = 1 and a difference between experimental and fitting *Q*_eq_ of less than 1%. Seven kinetic data sets for seven adsorbate concentrations and papaya seeds as adsorbent were presented in a work of Hameed [64], in which PS1, PS2, and INTD-1 were used to fit the data. The fitting quality was excellent for PS2 but quite unacceptable for PS1 according to the given *R*^2^ values. There was no linear relation between the experimental value of *Q* and *t*^1/2^, so the model INTD-1 could not describe the data. Seven kinetic data sets for seven adsorbate concentrations and spent tea leaves as adsorbents were presented in another work [123], where PS1, PS2, and INTD-2 were employed. PS1 gave an acceptable fit, while model (ii) an excellent one. Detailed fitting data (parameters and *R*^2^ for each region) were given to fit with multiple lines (two to three) of the experimental value of *Q vs. t*^1/2^ data.

Six sets of kinetic data for several adsorbate concentrations and rejected tea as adsorbent were presented in a study [124]. PS1, PS2, and INTD-2 were used. Only bilinear fitting was possible for *Q vs. t*^1/2^ graph, so INTD-2 was not acceptable. The fitting quality was poor for PS1 and acceptable for PS2. This was the only work where in addition to *R*^2^ the normalized standard deviation between model and experimental results was presented.

The adsorption of MB and Acid blue 25 (AB25) on hazelnut shells and wood sawdust was also studied [63]. In total, 21 kinetic data sets were presented (considering two particle sizes for hazelnuts and several adsorbate concentrations) and PS1 and PS2 were tested. Fitting results were presented for a total of 25 cases. The model PS2 led to excellent fitting results. The adsorption of dyes on prickly pear cactus cladodes was studied by Bark and co-workers [62]. Four sets of kinetic data for MB (four particle sizes) and two sets for two additional dyes were presented. PS1, PS2, ELV, and INTD-2 were used to fit three sets of data (for the smaller particle size). The fit was excellent for model PS2 quite good for model PS1 and acceptable for model ELV. INTD-2 could fit the data only locally in *Q vs. t*^1/2^ graph, so it was unacceptable. Three sets of kinetic data for MG adsorption by a low-cost activated carbon (for three temperatures) were presented by Uma and co-workers [72]. PS1, PS2, INTD-1, and MCK were employed. PS1 and INTD-1 gave unacceptable *R*^2^ values whereas for PS2, *R*^2^ = 1. The performance of MCK was shown graphically to be poor. The extracted h values exhibited negligible temperature dependence. A single data set for adsorption of Rhodamine *B* (on animal bone meal was also studied [73]. Several kinetic data sets for four temperatures and five adsorbate concentrations were fitted assuming an exponential relation between *C* and *t* (equivalent to the dynamics predicted by model PS1). The adsorption of disperse red and disperse blue dyes on palm ash was studied from another researcher [76]. Ten kinetic data sets (two dyes, five adsorbate concentrations) were presented. PS1 showed a very poor fitting quality (especially for the red dye). A very good fitting was achieved by PS2.

Twelve kinetic data sets for adsorption of three reactive dyes on wheat bran (four adsorbate concentrations) were fitted in a study [125]. PS1 and INTD-2 gave unacceptable fit. PS2 fits the data with constant *R*^2^ = 0.999.

Two data sets for adsorption of two direct red dyes by orange peels were shown in a numerical study [75]. PS1 and PS2 were applied to four data sets (two concentrations for each dye). Only model PS2 gave an acceptable fit.

Two kinetic data sets for adsorption of Congo red (CR) on two low-cost adsorbents were presented by Ghaedi and co-workers [126]. Detailed data for fitting using PS1, PS2, ELV, and INTD-2 were presented in tables. Only PS2 gave *R*^2^ values that correspond to successful fitting. Two sets of kinetic data for adsorption of a direct blue dye by two types of TiO_2_ were shown in another study with no modeling attempt [77].

Two sets of kinetic data for V54 adsorption by musa spp. waste were presented by Kumar and co-workers (two adsorbate concentrations) [74]. PS1, PS2, and INTD-2 were used but only PS2 gave acceptable fitting. Nevertheless, *R*^2^ for PS2 was smaller than 0.99 in all cases which was unusual. Six kinetic data sets for adsorption of a reactive black dye on sunflower seed shells (two pH values, three adsorbate concentrations) were presented in a study of Osma and co-workers [127]. PS2 and INTD-1 were applied only to data for pH = 2. PS2 gave an excellent fitting. The three slopes of tri-linear fitting on *Q vs. t*^1/2^ graph were given in tables (implying inability of INTD-1 to describe the data). The adsorption of a basic red dye on low-cost activated carbon and activated slag was also discussed in another study [128]. Eighteen kinetic data sets (two adsorbents, three different dosages for each one, three particle sizes, three temperatures) were presented. PS1 was applied to two data sets and only the two kinetic constants was given (fitting quality is acceptable according to the corresponding figure). The adsorption of a reactive and a basic blue dye on five chitosan derivatives at three temperatures was examined in a previous work of our research team [129]. In total, 11 kinetic data sets were presented. GREA was used to fit the adsorption dynamics for all possible combinations of dye-adsorbent-temperature. The procedure was to fit first the data assuming only pore diffusion. In case the values of pore diffusivity being smaller than bulk diffusivity, pore diffusion was the dominant adsorption mechanism; otherwise surface diffusion had to be considered. In this view, pore and surface diffusivities for all pairs of adsorbent-adsorbate were given and discussed in the context of adsorbent-adsorbate physicochemical interaction. The same procedure was used in our subsequent work employing ODGREA instead of GREA [130]. The two models were qualitatively equivalent since they exhibited the same parametrization but ODGREA is much simpler mathematically than GREA.

### 3.2. Metals—Ions

Five sets of adsorption kinetic data for adsorption of Cu(II) on five adsorbents were presented in a paper of Cochrane *et al*. [78]. The data were fitted employing the typical sequence of PS1, PS2, INTD-1 with their typical linear form. The fitting was successful as (according to the *R*^2^ values shown) only for PS2.

A single set of kinetic data for adsorption of Cd(II) on chemically modified olive stone was presented by Aziz and co-workers [82]. Authors stated that the model PS1 failed to fit the data (no results shown). PS2 successfully fitted the data giving *R*^2^ = 0.999. Several kinetic data for Cr(VI) adsorption on wollastonite (for several temperatures, pH values, ionic strengths and initial adsorbate concentrations) were presented by Sharma and co-workers [131]. The optimum adsorption conditions were found and the corresponding experiments were fitted with PS1, INTD-1, and MCK for three different temperatures. Although no explicit results for *R*^2^ were given, it is shown (in figures) that the three models successfully fitted the data and the corresponding parameters were given in the table for three temperatures. The fact was that three models based on different physical mechanisms are considered equally acceptable to describe the process. The fitting results showed a large increase of the mass transfer coefficient with temperature which according to the authors was related to the endothermic nature of the particular adsorption process.

A very extensive kinetic study of Cr(VI) adsorption on Alligator weed was published by Wang and co-workers [89]. Kinetic results for several temperatures, several adsorbent dosages and several initial adsorbate concentrations were presented. PS2 and ELV were fitted to the data. Both models were considered to be successful in describing the data, but *R*^2^ was clearly closer to unity for PS2. The model parameters were presented in tables with respect to the adsorbent and adsorbate quantities confirming the empirical nature of the models used.

Kinetic data for adsorption of Cu(II) by ash-based adsorbents was given by a study of Harja and co-workers [132]. The data included several adsorbents and adsorption parameters (pH, adsorbent’s dosage and adsorbate concentrations). PS1, PS2 and INTD-2 were fitted to the data referring to different adsorbents and different adsorbent dosages. The fitting results and the corresponding *R*^2^ values were presented in table form, revealing that only PS2 achieves successful fitting.

Kinetic data for the adsorption of Cd(II) on “wheat bran” at several temperatures and several adsorbate concentrations were presented by Singh and co-workers [133]. PS1, INTD-1, and MCK were fitted to a part of the data (one adsorbate concentration and three temperatures). No *R*^2^ results were given, but from the figure it was clear that INTD-1 and MCK failed to represent the data, while only PS1 could successfully describe them. Unlike the work of Sharma (discussed above [131]), h decreases as temperature increases.

Four kinetic data sets for As(III) and As(V) adsorption by natural red earth were given in another study (four combinations for controlled/uncontrolled pH, As(III)/As(V) adsorbate) [134]. The models PS1, PS2, and INTD-2 were fitted to the data. The *R*^2^ values shown in tables revealed that only PS2 could adequately describe the data.

A single set of kinetic data for adsorption of As(III) by Fe-based back washing sludge appeared by Wu and co-workers [135]. The data were fitted to ELV, INTD-1 and INTD-2. ELV seemed to achieve a good fitting and INTD-1 an acceptable one (with an exponent equal to 0.19 instead of 0.5). INTD-2 failed to describe the whole set of data, but it would have partial success if it had been applied in three different intervals as shown by the authors. Such a modeling approach lacked of any physical meaning. Four sets of kinetic data (different adsorbate concentrations) for Pb(II) adsorption by natural zeolite-kaolin-betonite combinations are presented in a paper of Salem and co-workers [136]. The data were fitted poorly to PS1 and successfully to model PS2.

Two data sets for U(VI) adsorption by two low-cost carbonaceous adsorbents were given by Liu and co-workers [137]. PS1 and PS2 were applied to the data, but only PS2 could successfully fit them. Kinetics data for Pb(II) and Cu(II) adsorption on tea leaves for four adsorbent dosages and four adsorbate concentrations (in total, 15 data sets) were presented by Amarsinghe and co-workers [138]. PS1 and PS2 were employed to describe the data for seven data sets (one adsorbent dosage), but results have been shown only for PS2, since it provided better correlation to the data according to the authors. Fitting results were also given for three additional data sets of Cu(II) adsorption with different adsorbent’s particle size. In total, 18 sets of kinetic data for adsorption of Cr(III) onto low-cost activated carbons were presented by Mohan and co-workers [139] (two types of adsorbents, three temperatures, three adsorbate concentrations). PS1, Ps2 and INTD were applied to all the data sets. The correlation coefficient *R*^2^ was shown only for PS1 (very poor fitting) and PS2 (successful fitting). The performance of INTD was shown only graphically to be of partial success. The resulting values of diffusion coefficients (presented as thermodynamics parameter) were of the order of 10^−16^ m^2^/s raising questions about their physical validity. Six sets of kinetic data for adsorption of Cu(II) and Cr(VI) on three chitosan derivatives are presented in [140]. The GREA with pore diffusion only was used to fit the data. The values of pore diffusivity discovered in the fitting procedure were about half of the diffusivity of the metals in water, so the resulted assumption was a sole pore diffusion process.

Some works presenting kinetic data of metal adsorption by low-cost adsorbents with no attempt to model/fit the data will be discussed in the following. Nine sets of kinetics data for adsorption of Cu(II), Pb(II), Ni(II) by Turkish lignites were presented by Pehlivan and Arslan [141]. Six sets of kinetic data for Cu(II) and Zn(II) adsorption by three low-cost adsorbents are presented by Omar and co-workers [79]. Some other adsorption kinetic data sets could be also found in literature: four sets for Cr(VI) adsorption by four low-cost activated carbons [142], nine sets for Cu(II) adsorption by Kolubara lignite (three adsorbent dosages and three adsorbate concentrations) [141], two data sets for adsorption of Pb(II) and Ni(II) by sawdust of lam tree [143] and five sets for Cr(VI) adsorption by four biological wastes and vermiculite [144].

### 3.3. Others

A significant amount of kinetic studies on other adsorbates (neither metals nor dyes) refers to phenolic compounds. Eight kinetic data sets for adsorption of 2-chlorophenol by coir pith carbon (four adsorbate concentration and four temperatures) are discussed by Namasivayam and Kavitha [88].

A total of twelve kinetic sets for adsorption of parachlorophenol and 2,4,6 trichlorophenol by activated carbon from coconuts shells (six adsorbate concentrations) are presented in another study [145]. In both the aforementioned works [88,145], PS1 and PS2 are fitted to the data. Only PS2 gives acceptable values of *R*^2^ coefficient. Five sets of adsorption data for four methylophenols on a low-cost carbonaceous adsorbent are presented by Jain and co-workers [90] (an additional concentration for one of the adsorbates is considered). PS1 is successfully used to describe the data as it is shown in graphical form (no *R*^2^ is given). In a similar work [91], four kinetic data sets for adsorption of three bromophenols on industrial waste adsorbents can be found. The model PS1 describes again adequately the data (*R*^2^ is given). The first order kinetic constant is related to the adsorbate molecule size.

Four sets of kinetic data for adsorption of a methyl parathion pesticide on four low-cost adsorbents are presented by Akhtar and co-workers [95]. PS1 and INTD-1 are used to fit the data. The fitting quality is very poor for both models. A total of 30 sets of kinetic data for pyridine adsorption by five low-cost activated carbons are presented (three adsorbent dosages, three temperatures) in a paper of Mohan and co-workers [92]. PS1 and INTD are considered, both of which fail to adequately represent the data as shown by the corresponding figures and tables. The diffusion coefficient found from fitting ranges from 10^−10^ to 10^−8^ m^2^/s. The last one is too large to have any physical meaning. A single kinetic data set (fitted by PS2) for cyanine acid adsorption by a CuFe_2_O_4_/sawdust nanocomposite was also presented in other work [94]. Furthermore, two data sets for fluorides adsorption on betonite and charfines were discussed by Srimurali and co-workers [146] and two data sets for nitrobenzene adsorption by two crop biological wastes can be found in another study [93]. There are no kinetic modeling attempts in the last two works.

## 4. Techno-Economical Analysis and Future Aspects

As it is said previously, the most used adsorbent material is activated carbon. The world demand for virgin activated carbon is forecasted to expand 5.2 percent per year through 2012 to 1.15 million metric tons. The consumption of activated carbons for industrial use has now become an indicator of development and environmental management efficiency. The per capita consumption of activated carbons per year is 0.5 kg in Japan, 0.4 kg in the U.S., 0.2 kg in Europe, and 0.03 kg in the rest of the world [147]. After the adsorbents are exhausted, they are either to be disposed off or regenerated for use. This depends upon the demand, economics involved, and the kind of pollutant that was adsorbed. In many cases, spent adsorbents are to be treated as hazardous waste and need to be incinerated (which in many countries causes a set of environmental and societal problems) [148]. Exposure of spent adsorbents to ambient air may result in accumulation of heat due to adsorption of moisture and desorption of toxic adsorbates, creating hazardous conditions. Dumping spent adsorbents may also cause odour resulting in nuisance. The other option that industry can use is regeneration. Regeneration costs may equal to stabilization costs or slightly more than that, but if consumption of virgin adsorbent is reduced then multiple economic, industrial and environmental benefits can be gained. Extensive research has already been conducted regarding adsorption of pollutants onto various activated carbons, but investigations into regeneration remain scarce [149]. In many cases, the adsorbates may be a resource and need to be recovered or concentrated to earn recovery credits. Considering all above arguments it is evident that spent adsorbent needs to be stabilized after being discarded. Because of high costs of production, stabilizing or proper disposal seem unwilling operations. Regeneration of adsorbents could prove double rewarding by stabilizing adsorbents and recovering valuable adsorbates, thereby minimizing demand for virgin adsorbents.

The main drawback of the already published adsorption studies is that their use is still in the laboratory stage mostly without pilot studies or commercialization. Limited attempts for detailed economic and market analyses are available [150]. Some attempts have been realized in the past at commercializing immobilized biomass dye biosorbents such as alga_SORB, AMT-bioclaim, B.V. Sorbex’s biosorbents and Bio-fix, but none have made a successful commercial entry in the market [151–153].

The main concept is not to extensively study the various fixed-bed adsorption papers in literature (and many of the parameters such as flow rate, bed volume, cross-sectional area, length, void fractions, adsorbent’s density, approach velocity, effective contact time, empty bed contact time, operation time, throughput volume, specific throughput, bed volumes), but analyze and evaluate the first and fundamental principles of the use of green adsorbents.

The first topic is to decide whether any modification for the green adsorbent is required. Low cost adsorbents can be modified before being used for the removal of pollutants. The modification can increase the adsorption capacity of the materials. There are different modification methods and these can be done using various agents like base solutions (sodium hydroxide, calcium hydroxide, sodium carbonate) mineral and organic acid solutions (hydrochloric acid, nitric acid, sulfuric acid, tartaric acid, citric acid, thioglycollic acid), organic compounds (ethylenediamine, formaldehyde, epichlorohydrin, methanol), oxidizing agents (hydrogen peroxide) and dye (Reactive Orange 13), *etc.* The chemical modification can increase the number of active binding sites in the material, improve the ion exchange properties and form new functional groups that favor metal uptake [54]. However, for some possible modifications in green adsorbent use, the cost will be drastically increased.

In the case of use some green materials (mainly wastes) as sources for production of activated carbon, there is one serious problem: the regeneration cost. The costs of activated carbon adsorption are relatively high and the high costs limit its use in large-scale applications. The investment costs consist of the costs of equipment (contactors, pumps, pipes and monitoring systems). The operational costs depend mainly on the price of the adsorbent. The costs are reduced when the adsorbent consumption per unit volume of treated wastewater is lowered. In the adsorption process, electricity is mainly used for pumping the water and mixing the adsorbent suspension and for regeneration. In addition, the costs of regeneration and reactivation and the disposal costs of spent adsorbent must be taken into account when the total costs of adsorption are estimated. Spent adsorbent can include toxic substances and they have to be treated as hazardous waste. The main conclusion from the above is that if an adsorbent is low cost but difficult to regenerate, it could not be economical and attractive for use [154,155].

As discussed above, the most important parameter determining the cost of the adsorption process to scale it up is the adsorbent and regeneration costs. Furthermore, in a hypothetical scenario of using a green adsorbent produced from wastes, some other costs need to be taken into consideration. The cost for the adsorbent waste treatment consists of the cost of dewatering, transport and treatment by incineration or landfill and is estimated at 100 €/ton [155]. The adsorbent concentration needed to comply with the imposed discharge limits is influenced by the initial concentration of the pollutants in the water and the pollutant removal ability of the adsorbent. In systems containing only one pollutant and one adsorbent, an adsorption isotherm relates the adsorbents’ capacity to the pollutant concentration in the water at equilibrium conditions. Therefore, the appropriate adsorbent’s dosage has to be determined. For this reason, Kyzas and co-workers [156–158] studied the use of spent coffee wastes for dyes and heavy metal removal. It was shown that 5 g/L was the best adsorbent’s dosage for the full decolorization of dyeing effluents. However, in each case, it is necessary to determine the environmental limits/regulations (discharge to aquatic systems) for each pollutant.

Another crucial factor regarding the operating cost of simulating adsorption procedure is the electricity, which is mainly used for pumping the water and mixing the adsorbent suspension. Vreysen and co-workers made a very useful cost estimation of the electricity required for an adsorption-flocculation system. The main equations used are the following: The suspension is mixed for 15 min at *G* = 821 s^−1^. The energy dissipation can be calculated from the formula:

$$G={\left(\frac{P}{V\mu }\right)}^{1/2}$$ (24)

where *G* is the average velocity coefficient, *V* is the water volume, μ is the dynamic viscosity of water (8.9 × 10^−4^ Pa·s) and *P* is the power required (Watt).

For the flow rate:

V=QT (25)

where *Q* is the flow rate (m^3^/h) and *T* is the time (h). Combining Equations (24) and (25) results in:

$$P=G^{2}QT\mu [W]$$ (26)

or

$$\frac{P}{Q}=\frac{G^{2}T\mu }{1000}[kWh/m^{3}]$$ (27)

**Figure 2.**
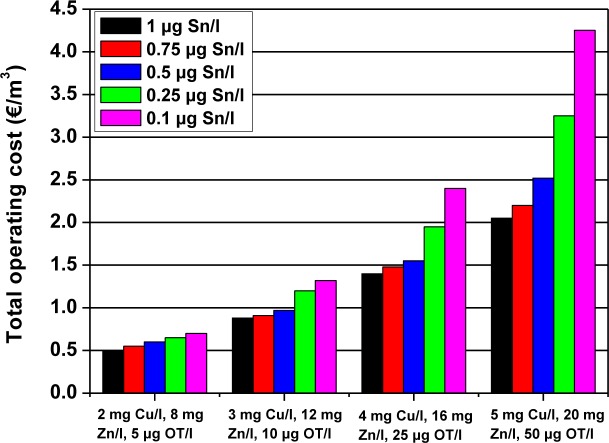
Total operating costs for four different wastewaters for five different organotin discharge limits. Reprinted with permission; Copyright Elsevier (2008) [155].

The total operating cost consists of the sum of the adsorbent cost (including sludge treatment) and the electricity cost. Figure 2 shows a total operating cost estimation for four polluted wastewaters and five different organotin discharge limits as done by Vreysen *et al*. [155]. The applied discharge limits for Cu and Zn were taken as 0.5 mg/L Cu and 2 mg/L. Zn in all cases.

From all above, it is clear that the most profitable use of green adsorbents is not those derived from activated carbon, but from agricultural wastes. The use of those wastes untreated (just washed) seems to be even better. A possible scenario used to make a comparison is as follows:

The parameters hypothetically are the same apart from the maximum theoretical adsorption capacity (*Q*_m_) and the estimated cost for the adsorbent production. In that case, 10, 5, and 3.3 kg of AW, AC, and ACM required for decontamination. However, in the case of AW the production cost estimated to be zero. Instead, a factor of 0.5 is added. The production of AC is expected to be at least four times larger (electricity for pyrolysis *etc.*), while the respective for ACM is six times larger (pyrolysis, chemical modification *etc.*). It is clear that the order of profitability using the above adsorbents will be ACM < AC < AW. In another scenario, in which both other parameters vary, the superiority of green adsorbents will be even clearer.

In order to make a more realistic scenario, for an average industry which treats and discharges 1 MGD (megagallons per day) as effluents (containing either dyes or metals), the approximate quantity of adsorbents can be calculated. In the case of textile industries, dye concentrations of 0.01–0.25 g/dm^3^ (=10–250 g of dye per m^3^ of effluent) have been cited as being present in dyehouse effluents, depending on the dyes and processes used [35]. Therefore, 37.85–946.25 kg of dye (containing the dyeing effluent) per day must be removed/adsorbed. Having as a basis the example of Table 1, 378.5–9462.5 kg of AW, 189–4731 kg of AC and 126–3154 kg of ACM are needed for the efficient treatment of effluent. However, as explained in the previous paragraph, the cost for the synthesis of AW is nearly zero. So, in any case, this process can be characterized as sufficient. The same example for an average metal plating (chromium) with 2 MGD as the effluent rate can be calculated mentioning that chromium concentrations of 0.5–270,000 g per m^3^ of effluent have been cited [159].

The future aspects are clear. The main future target of market is to move the adsorption process to an industrial scale. It is relatively less difficult to demonstrate it in a laboratory; it is a little more challenging to demonstrate it at a pilot scale, but to really scale it up to a large scale would call for a significant financial and technological effort. This mismatch between scientific progress in biosorption research (biosciences) and stagnation in industrial technology innovation needs to be corrected through translational research and technology transfer with a push for commercialization of research. Universities can play an active role in this process through more formalized approach to technology transfer and protection of intellectual property [150,160].

## 5. Conclusions

This review is a critical approach to green adsorption. Many different (maybe in some occasions doubtful) topics are discussed as: (i) adsorption capacity; (ii) kinetic modeling (given the ultimate target to scale up the batch experimental data to fixed-bed column calculations for designing/optimizing commercial processes) and (iii) critical techno-economical data of green adsorption processes in order to scale-up experiments (from lab to industry) with economic analysis and perspectives of the use of green adsorbents. Three isotherm models are given in recent literature in order to fit the experimental equilibrium data: the Langmuir, Freundlich and the combinational Langmuir-Freundlich (L-F) equations. The majority of research works presented very low adsorption capacities (especially for the case of phenols and dyes) due to the non-modified use of these adsorbents. Lagergren and pseudo-second order equations, along with Elovoich, Intraparticle and McKay models, are used to fit the kinetics of low-cost adsorbents. Also, chemical reaction engineering approach (GREA) and an ordinary differential GREA were also fitted to data. A techno-economical analysis was also done comparing directly a green adsorbent material with a non-green one. The major conclusion was the superiority of effectiveness of green adsorbents (cost of synthesis, regeneration *etc.*) regarding commercial super-adsorbents.

## Figures and Tables

**Table 1.** Techno-economical comparison for green and non-green adsorbents.

Parameter	Green adsorbents		Non Green adsorbent
Adsorbent	Agricultural wastes (AW)	Activated carbon (AC)	Activated carbon (ACM)
Pollutants used	Dyes, Metals, Others	Dyes, Metals, Others	Dyes, Metals, Others
Modification	No	No	Yes
Adsorption capacity	100 mg/g	200 mg/g	300 mg/g
Mass of pollutant for removal	1 kg	1 kg	1 kg
Adsorption-desorption cycles	20	20	20
Loss of capacity after cycles	20%	20%	20%
Estimated cost for the adsorbent production *	0.5	2	3
Mass of adsorbent required	10 kg	5 kg	3.3 kg
Order of profitability	1	2	3

* This factor is used instead of using exact/unknown prices.
